# Ki-67 proliferation index but not mitotic thresholds integrates the molecular prognostic stratification of lower grade gliomas

**DOI:** 10.18632/oncotarget.8498

**Published:** 2016-03-30

**Authors:** Eleonora Duregon, Luca Bertero, Alessandra Pittaro, Riccardo Soffietti, Roberta Rudà, Morena Trevisan, Mauro Papotti, Laura Ventura, Rebecca Senetta, Paola Cassoni

**Affiliations:** ^1^ Department of Medical Sciences, University of Turin, Turin, Italy; ^2^ Department of Oncology, University of Turin, Turin, Italy; ^3^ Department of Statistical Sciences, University of Padua, Padova, Italy; ^4^ Department of Neuro-Oncology, University and City of Health and Science Hospital of Turin, Turin, Italy; ^5^ Cancer Epidemiology Unit-CERMS, Department of Medical Sciences, University of Turin, Turin, Italy; ^6^ CPO-Piemonte, Turin, Italy

**Keywords:** lower grade gliomas, Ki-67 index, phospho-histone H3, prognosis, Pathology Section

## Abstract

Despite several molecular signatures for “lower grade diffuse gliomas” (LGG) have been identified, WHO grade still remains a cornerstone of treatment guidelines. Mitotic count bears a crucial role in its definition, although limited by the poor reproducibility of standard Hematoxylin & Eosin (H&E) evaluation. Phospho-histone-H3 (PHH3) and Ki-67 have been proposed as alternative assays of cellular proliferation. Therefore in the present series of 141 LGG, the molecular characterization (namely IDH status, 1p/19q co-deletion and MGMT promoter methylation) was integrated with the tumor “proliferative trait” (conventional H&E or PHH3-guided mitotic count and Ki-67 index) in term of prognosis definition. Exclusively high PHH3 and Ki-67 values were predictor of poor prognosis (log rank test, *P* = 0.0281 for PHH3 and *P* = 0.032 for Ki-67), unlike standard mitotic count. Based on Cox proportional hazard regression analyses, among all clinical (age), pathological (PHH3 and Ki-67) and molecular variables (IDH, 1p/19q codeletion and MGMT methylation) with a prognostic relevance at univariate survival analysis, only IDH expression (*P* = 0.001) and Ki-67 proliferation index (*P* = 0.027) proved to be independent prognostic factors. In addition, stratifying by IDH expression status, high Ki-67 retained its prognostic relevance uniquely in the IDH negative patient (*P* = 0.029) doubling their risk of death (hazard ratio = 2.27). Overall, PHH3 immunostaining is the sole reliable method with a prognostic value to highlight mitotic figures in LGG. Ki-67 proliferation index exceeds PHH3 mitotic count as a predictor of patient's prognosis, and should be integrated with molecular markers in a comprehensive grading system for LGG.

## INTRODUCTION

Lower-grade diffuse gliomas (LGG) (World Health Organization -WHO- grade II and III diffuse gliomas) are diffusely infiltrative primary glial brain tumors including astrocytomas, oligodendrogliomas, and mixed oligoastrocytomas. LGG bear a highly variable clinical behavior, as some of them rapidly progress to glioblastoma (WHO grade IV gliomas), whereas others and have a remarkable therapeutic sensitivity, remaining stable for years [1]. Current treatment options are driven by the extent of resection, histotype, tumor grade, and the results of ancillary testing. Several molecular signatures have now been identified (IDH, 1p/19q co-deletion, ATRX, TERT, p53, MGMT promoter methylation), with important diagnostic, prognostic, and predictive roles. This allows further stratification of gliomas into several distinct subgroups [2]. Nevertheless, both WHO grade and glioma morphology remain two cornerstones of treatment guidelines and ongoing clinical trials [3, 4]. Glioma grading is based on the definition of few morphological features, including mitotic count, observation of increased cellularity with nuclear atypia, necrosis and microvascular proliferation. The diagnostic reproducibility of morphologic criteria for LGG is still influeced by a considerable inter-observer variability among pathologists [5, 6]. One of the major determinants of such variability concerns the proliferative activity, as WHO grading criteria does not define strict mitotic figure cut-offs to distinguish grade II from grade III tumors. Mitotic count is based on the observation of the number of mitoses per 10 high power fields (mitoses/10HPF) on standard Hematoxylin and Eosin (H&E) stained slides. The accuracy of this method can be influenced by both technical artifacts and experience of the pathologist performing the count. To overcome these inconveniences, phospho-histone-H3 (PHH3) and MIB1/Ki-67 have been proposed as alternative assays of cellular proliferation. PHH3 is a mitosis-specific antibody, which highlights the cell nucleus during the late-G2 and all mitotic stages, but negligibly at any other time (including apoptosis) [7, 8]. Conversely, Ki-67 is the most reliable marker of cell proliferation, which stains cells in all phases of cell cycle except G0. In LGG, Colman et al. [9] compared the prognostic significance of standard H&E-based mitotic count (number of mitoses per 10 HPF), PHH3-based mitotic index (number of mitoses per 1000 cells), and Ki-67 proliferation index (number of positive nuclei per 1000 cells), identifying prognostic cut-off for each method (≤3 *versus* > 3/10 HPF for H&E, ≤ 4 *versus* > 4/ 1000 cells for PHH3 and ≤ 9 *versus* > 9 for Ki-67) and proposing PHH3 as the most useful and clinical relevant proliferation marker. The same cut-off for PHH3 was recently [2] adopted by the same group to demonstrate a PHH3 prognostic role only in specific subsets defined by the presence or absence of IDH mutation.

However, to the best of our knowledge in LGG a) the inter-observer reproducibility of PHH3 has never been investigated, b) the initially proposed PHH3 thresholds [9] were not further validated by other groups, c) an exhaustive comparison of proliferation assays as well as d) the meaning of their integration with prognostic and predictive relevant molecular markers are still lacking.

Therefore, a comprehensive morphological and molecular study on a series of 141 LGG was designed to evaluate the reliability of different methods to assess proliferation and to define their effect in modulating the prognostic role of molecular characterization.

## RESULTS

### Clinicopathological features

**Figure 1:**
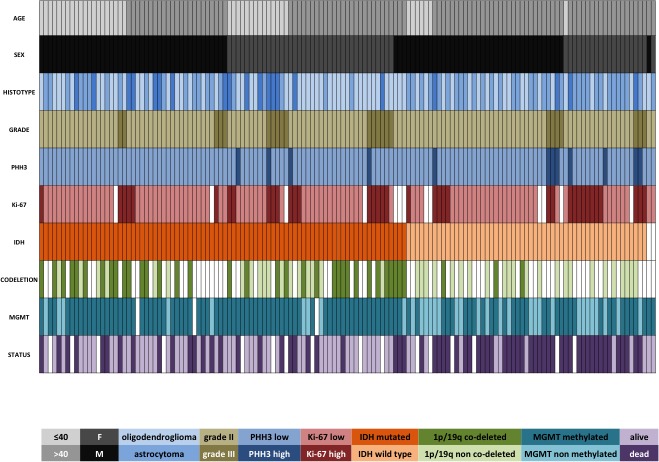
Clinical and molecular data regarding the whole cohort of 141 LGG Each column represents a patient. In two patients with wild type IDH and lacking 1p/19q co-deletion, the tissue was insufficient for IDH direct sequencing.

**Figure 2:**
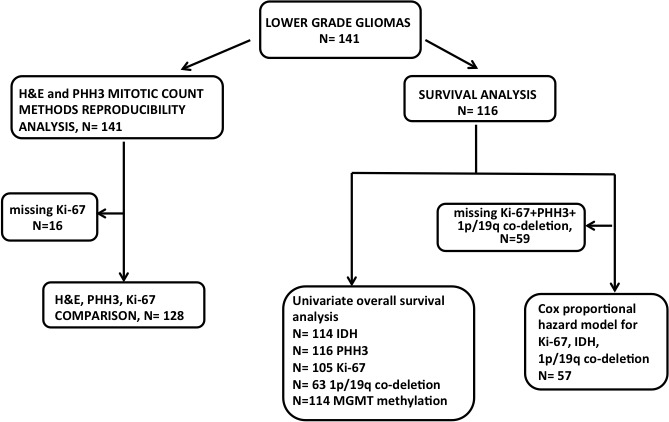
Diagram illustrating analysis strategy and number of patients included at each step Given that some samples had missing clinical or molecular data, fewer patient samples were available for Cox-Proportional Hazards analysis.

The clinical and pathological features of all cases are summarized in Table 1 and Figure 1. There were 83 males and 58 females, having a median age of 50 Years (range 19-81). Most patients received chemotherapy only, based on temozolomide, an alkylating agent. The series accounted for 67 oligodendrogliomas, 55 astrocytomas, and 19 mixed oligo-astrocytomas. According to WHO histological grading, 118 cases (84%) were grade II, while the remaining 23 (16%) were classified as grade III. In the study 81 of 139 cases (58%) were IDH mutated, among which the majority were oligodendrogliomas (41, 29%). Co-deletion of 1p19q was present in 37 of 79 tumor tested (47%), 34 of which were IDH mutated. Finally, MGMT promoter methylation was a feature of 105 out of 139 cases analyzed (75%). A diagram of the complete analytical strategy, and the flow of patients through the study, including the number of patients included at each stage of the analysis, is shown in Figure 2.

**Table 1:** Clinicopathological features of the whole series

		All patients	IDH Mutation	1p/19q co-deletion	MGMT methylation
**Age**	**≤40** **>40**	41 100	34 44	14 23	34 71
**Sex**	**female** **male**	58 83	36 42	15 22	46 59
**Treatment**	**observation** **chemotherapy (TMZ)** **RT alone** **RT+TMZ** **not available**	31 57 11 22 20	22 30 8 10 8	8 19 3 1 6	25 43 8 15 14
**Histotype**	**Oligodendroglioma** **Astrocytoma** **Oligoastrocytoma**	67 55 19	41 22 15	29 2 6	50 37 18
**WHO grade**	**II**	118	62	30	84
	**III**	23	16	7	21

*Abbreviations:* RT, radiotherapy; TMZ, temozolomide.

### Correlation and reproducibility of mitotic count methods: H&E compared to PHH3

**Figure 3:**
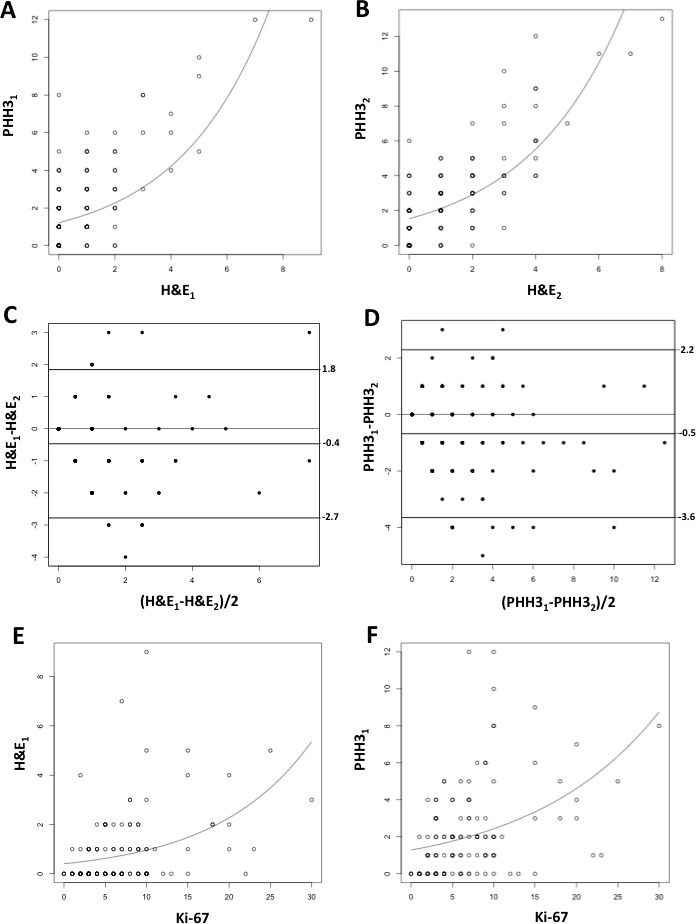
Quasi-Poisson regression model for PHH3 *versus* H&E mitotic counts for Observer 1 [PHH3 = exp(0.62+0.14xH&E)] **(A) and for Observer 2 [PHH3 = exp(0.66+0.26xH&E)] (B).** Bland-Altman plots demonstrating the mean difference between mitotic counts produced by two observers (H&E_1_ and PHH3_1_ for Observer 1; H&E_2_ and PHH3_2_ for Observer 2) based on H&E (−0.468, 95% confidence interval = −2.78, 1.84) **C.** and on PHH3 stained slides (0.68, 95% confidence interval = −3.65, 2.28) (**D)**. Quasi-Poisson regression model for Ki-67 index *versus* H&E [**E**, H&E = exp(−0.18+0.07xKi67)] and PHH3 mitotic count [**F**, PHH3 = exp(0.75+0.07xKi-67)].

Manually counted mitoses had a median value of 1 /10 HPF (range 0-8) on H&E-stained slides. All cases were evaluable for the PHH3 antibody, which allowed for a more straightforward and swift count as highlighted. With the PHH3 antibody, the median mitotic count was of 2 mitoses/10 HPF (range 0-13). A quasi-Poisson regression model for PHH3 *versus* H&E mitotic counts showed a good fit in particular for small counts (Figure 3A). The fitted model was PHH3 = exp(0.62+0.14xH&E), with residual deviance 451.9 (df = 157, dispersion parameter = 3.03). After evaluating the same set of slides, another observer produced a mean value of 0 (range 0-9) and 1 (range 0-12) mitoses/10 HPF for H&E-based and PHH3 mitotic count, respectively. As for the first observer, a quasi-Poisson regression model showed a good association for PHH3 *versus* H&E mitotic counts (PHH3 = exp(0.66+0.26xH&E), residual deviance 281.3, df = 159, dispersion parameter = 1.61) (Figure 3B). H&E mitotic counts were found with a high internal consistency between observers (alpha cronbach = 0.82) and the same happened for mitotic counts evaluated on PHH3 stained slides (alpha cronbach = 0.906). Bland-Altman plots demonstrated a high degree of agreement both for H&E and for PHH3 mitotic counts (H&E mean difference: −0.468, 95% confidence interval = −2.78, 1.84 Figure 3C; PHH3 mean difference: −0.68, 95% confidence interval = −3.65, 2.28 Figure 3D).

### Comparison between H&E/PHH3 mitotic counts and Ki-67 proliferation index

Ki-67 proliferation index had a median value of 6% (range 0-30%). The estimated quasi-Poisson regression models for H&E and PHH3 and the covariate Ki-67 were, respectively, H&E = exp(−0.18+0.07xKi-67) and PHH3 = exp(0.75+0.07xKi-67). The residual deviances for the two fitted models were, respectively, 271.2 (df = 142, dispersion parameter = 2.2) (Figure 3E) and 453.05 (df = 142, dispersion parameter = 3.68) (Figure 3F). Both quasi-Poisson regression models showed a good fit in particular for small counts. In both cases, the Spearman's correlations (r = 0.40 and 0.45, for Ki-67 against H&E and PHH3, respectively) were lower than that of PHH3 with H&E (r = 0.57).

### Correlation between clinico-pathological variables and proliferation measures

Both mitotic count methods and Ki-67 proliferation index were significantly associated with the histological diagnosis, having mixed oligo-astrocytic tumors higher values compared to oligodendrogliomas and astrocytomas (Kruskal-Wallis test, *P* = 0.002 for H&E, *P* = 0.005 for PHH3 and *P* = 0.039 for Ki-67). The significant association between high mitotic count and elevated histological grade (grade III) on H&E (Wilcoxon test, *P*= 0.018) was remarkably increased when using PHH3 (Wilcoxon test, *P* = 0.0002) and Ki-67 (Wilcoxon test, *P* < 0.0001). MGMT promoter methylation was related to high mitotic counts and PHH3 values (Wilcoxon test, *P* = 0.025 for H&E, *P* = 0.008 for PHH3, but not with Ki-67 values (Wilcoxon test, *P* = 0.279). Conversely, IDH mutation was not found to be associated with mitotic counts (Wilcoxon test, *P* = 0.271 for H&E, *P* = 0.135 for PHH3) or with Ki-67 index (*P* = 0.782) and the same occurred for 1p/19q co-deletion (Wilcoxon test, *P* = 0.619 for H&E, *P* = 0.825 for PHH3 and *P* = 0.642 for Ki-67).

### PHH3 and Ki-67-specific thresholds based on overall survival

As expected, at univariate survival analysis, both IDH mutation, 1p/19q co-deletion and MGMT methylation were all related to an improved survival (univariate Cox regression, *P* < 0.001 for IDH mutated, *P* = 0.001 for 1p/19q co-deleted and 0.008 for MGMT methylated cases Table 2). With respect to the three methods for assessing proliferation, only high PHH3 and Ki-67 values were significantly associated with a poor outcome (univariate Cox regression, *P* = 0.0281 for PHH3 and *P* = 0.032 for Ki-67, Table 2). Cut-off points specific for PHH3 and Ki-67, which divided cases into different groups based on their prognosis, were selected on the basis of the highest statistical significance on the log-rank test (lowest P values). The optimal cut-off was ≤ 6 and ≥ 7 for PHH3, and < 8.5% and ≥ 8.5% for Ki-67.

**Table 2:** Univariate analysis for overall survival

	HR	95% CI	*p*-value
**Age°**	1.03	1.01-1.05	***0.004***
**Histological diagnosis** **oligodendrogliomas *vs* astrocytomas** **oligodendrogliomas *vs* oligoastrocytomas**	2.63 2.94	0.79-2.68 0.55-3.79	0.22 0.499
**WHO grade (III *vs* II)**	1.11	0.49-2.46	0.806
**H&E mitotic count °**	1.10	0.91-1.31	0.331
**PHH3 mitotic count °**	1.11	1.01-1.23	***0.028***
**Ki-67 proliferation index °**	1.05	1.01-1.10	***0.032***
**IDH status** **(wild type *vs* mutant)**	3.66	2.04-6.66	***<0.001***
**1p/19q co-deletion** **(non co-deleted *vs* co-deleted)**	2.99	1.23-7.14	***0.001***
**MGMT methylation** **(non methylated *vs* methylated)**	2.12	1.2-3.77	***0.009***

Abbreviations: HR, hazard ratio; CI, confidence interval.

° Quantitative variable

*significant parameters at multivariate analysis: Ki-67: HR = 2.28 (95% CI 0.44-1.22), *P* = 0.001; IDH status: HR = 4.36 (95% CI 3.32-8.18), *P* = 0.0001.

**Figure 4:**
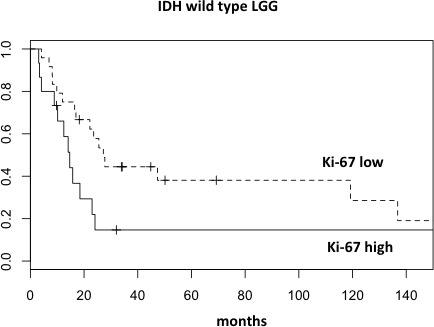
Overall survival curves of wild type IDH LGG segregated into two prognostic groups according to Ki-67 proliferation index

Cox multivariate analysis using a backward stepwise selection was performed based on these thresholds and on all other parameters with a statistical significance at univariate survival analysis. Within a restricted cohort (57 cases) for whom all co-variates were available, only IDH mutation status and Ki-67 proliferation index were statistically significant (Table 2). In addition, the Cox model applied to the same subset stratified by IDH mutation status showed a significant difference according to Ki-67 expression in the IDH wild type group (*P* = 0.034), but not in the IDH mutated (*P* = 0.197). As a matter of fact, IDH wild type patients with high Ki-67, had a doubled risk of death (hazard ratio = 2.27, 95% confidence interval 1.06-4.76) compared to those with low Ki-67 (Figure 4).

## DISCUSSION

We here, show that a) H&E and PHH3-guided mitotic count have a good intra-observer reliability and inter-observer reproducibility, especially for lower values; b) high PHH3 (but not H&E based) mitotic count and Ki-67 proliferation index are predictors of poor prognosis; c) at multivariate survival analysis, only Ki-67 proliferation index and IDH status retain independent prognostic value; d) high Ki-67 values identify a poor prognosis subset of patients within IDH wild type tumors.

### Mitotic count: PHH3 does not increase intra and inter-observer agreement

Mitosis recognition is a major factor responsible for the poor diagnostic reproducibility within LGG. This is due to the ambiguity of current WHO [10] in distinguishing grade II from grade III neoplasms (based on a “brisk mitotic activity”) and the lack of a definite mitotic figures cut-off. In addition, pathologist's individual expertise significantly influences the accuracy of diagnosis. Thanks to an immunohistochemical positivity matched to consistent morphologic features, PHH3 expression allows a rapid and selective recognition of all mitoses, therefore limiting under- or over-estimation. The great value of PHH3 has already been demonstrated in several human tumors in which mitotic count is crucial for diagnostic and/or prognostic purposes (i.e. melanoma [11], breast cancer [12], gastrointestinal stromal tumor [13], adrenocortical carcinoma [14] and meningioma [15]). In LGG, two previous reports confirmed the diagnostic usefulness of PHH3 [9, 16], although they did not investigate the concordance among pathologists. Therefore, the first aim of this study was to compare H&E and PHH3 intra and inter-observer reproducibility. A high concordance between morphologically and PHH3-based mitotic counts was detected, resulting in a good intra-rater reliability, especially for lower values. In addition, both H&E and PHH3-based mitotic count proved to have an excellent inter-observer agreement. Therefore, although PHH3 staining represents a useful tool to simplify mitosis screening in LGG, unlike other brain neoplasms, such as meningiomas [15], it does not perform any better than standard H&E assessment in terms of inter-observer reproducibility.

### Setting of specific PHH3 and Ki-67 thresholds in LGG

A comparison between the prognostic performance of mitotic counting (either H&E or PHH3 based) and Ki-67 proliferation index proved that exclusively PHH3-based mitotic count and Ki-67 proliferation index were associated to a greater risk of death at univariate survival analysis. Therefore, although the reproducibility of the two mitotic count methods is comparable, we discourage from using standard H&E slides in favor of PHH3 immunohistochemistry. For PHH3 mitotic count and Ki-67 proliferation index, specific cut-offs based on the highest statistical significance (lowest P values) on the overall survival were proposed. For LGG, a PHH3-specific threshold of 4 mitoses per 1000 cells had already been proposed [9] and subsequently confirmed [16]. However, this cut-off referred to a mitotic index, and not to a true mitotic count, which according to the WHO has to be performed in 10 consecutive HPF within the area of highest mitotic activity. We identified ≤ 6 and ≥ 7 mitoses/10 HPF as the best cut-off for PHH3 mitotic count. With respect to Ki-67, two independent groups introduced similar thresholds: 8% for Schiffer et al. [17] and 9% for Colman et al. [9]. Accordingly, in the present work we identified < 8.5% and ≥ 8.5% as the optimal cut-off with the highest statistical performance for Ki-67. Therefore, it can reasonably be concluded that the values of Ki-67, around 8 and 9%, are those that best discriminate two groups associated to a significantly different prognosis.

### Prognostic impact of a Ki-67-based stratification within the distinct molecular subclasses of LGG

The discovery of the mutations in the *IDH1* and *2* genes and the consistent deletion of chromosomal arms 1p and 19q represent to date the major diagnostic breakthrough in LGG classification, which bears prognostic impact. At this regard, the debate to what extent to integrate (or rely on) molecular data has become more and more underpinned [18, 19]. The integration of genome wide data by various groups has delineated different prognostic classes, which were more concordant with IDH and 1p/19q statuses than with histologic classifications [2]. As a consequence, the third and final purpose of this work focused on the use of PHH3 and/or Ki-67 as a complementary tool to molecular diagnosis in LGG prognostication. Based on Cox proportional hazard regression analyses, our data shows that among all clinical (age), pathological (PHH3 and Ki-67), and molecular variables (IDH, 1p/19q co-deletion and MGMT promoter methylation) bearing prognostic impact at univariate survival analysis, only IDH mutation and Ki-67 index were independent prognostic factors. Therefore, this is the first study demonstrating the Ki-67 superiority to mitotic count (irrespective of the method of estimation, standard H&E or PHH3-guided) in terms of prognostic stratification. In addition, after IDH stratification, Ki-67 retained its prognostic value solely in the IDH wild type patients. This finding is consistent with previous data [16] reporting that prognostic relevance for PHH3 mitotic index was limited to the cohort of *IDH* wild type LGG. The latter observation broadens the potential tools available to define patients' risk stratification and is strong enough to point towards a molecular-proliferative integrated and comprehensive grading system in LLG.

## MATERIALS AND METHODS

### Tissue collection

141 gliomas were retrieved from the pathology files of the University of Torino. All patients were resected at the Neurosurgery Unit of City of Health and Science Hospital of Turin (Molinette) between 2005 and 2014. The treatment of patients consisted of maximal safe resection, followed by observation or postoperative radiotherapy, and/or chemotherapy, depending on the histology, grade and extent of resection. For all cases, the clinico-pathological data were obtained and analyzed. The study received ethical approval from the local Review Board of our Institution.

### Immunohistochemistry, fluorescence *in situ* hybridization and molecular analyses

All available H&E stained slides were reviewed, and a representative paraffin block was selected for each case. Three μm thick serial paraffin sections were processed by immunohistochemistry using an automated immunostainer (Ventana BenchMark AutoStainer, Ventana Medical Systems, Tucson, AZ, USA) with antibodies against PHH3-Ser10 (Cell Marque-Roche, rabbit polyclonal antibody, prediluted, 0.03 μg/ml, Rocklin, CA, USA), Ki-67 (Dako, mouse monoclonal antibody, clone MIB-1, dilution 1/100, Glostrup, Denmark) and IDH1 R132H mutation (Dianova, monoclonal antibody, clone H09, dilution 1/20, Hamburg, Germany). A biotin-free, dextran chain-based detection system (EnVysion, Dako) and diaminobenzidine as the chromogen (Ventana Medical Systems, Tucson, AZ, USA) were used according to standard protocols. Appropriate positive controls were included. In case of IDH1 negative immunohistochemistry, IDH1/IDH2 mutations were further investigated by direct sequencing. IDH1 codons 100 and 132 and IDH2 codon 172 were evaluated according to standardized procedure. The tissue of two cases was not sufficient for IDH1/IDH2 molecular analysis. 1p/19q chromosomal arms co-deletion status was assessed by fluorescence *in situ* hybridization (FISH) using probes against 1p36/1q25 and 19q13/19p13 (Abbott Molecular, North Chicago, IL, USA). Methylation MGMT status was assessed by pyrosequencing. The extracted DNA was subjected to polymerase chain reaction (PCR) amplification with a forward primer and a biotinylated reverse primer using the “MGMT plus” kit (Diatech Pharmacogenetics, Jesi, Ancona, Italy); 10 CpG sites in the following regions: chromosome 10: 131,266,507-131,265,556 were evaluated).

### Morphological and PHH3-based mitotic index assessment

To assess the reproducibility of the mitotic count evaluation, all H&E and PHH3 stained slides were evaluated by two pathologists (AP, ED) who were separately asked to manually count mitoses in 10 consecutive HPF (each = × 400), both on H&E and PHH3 stained sections using the same microscope, with HP field set at the dimension of 0.16 mm^2^. PHH3-labelled mitotic figures were considered in the presence of a positive staining and consistent morphologic features [20]. The Ki-67 proliferation index was determined counting 1000 cells in hot spots and calculated as the percentage of positive nuclei by one senior pathologist (RS).

### Statistical analysis

Statistical analyses were performed using the free software R (http://www.r-project.org/). A significance level of 0.05 was used. Non parametric tests (Wilcoxon-Mann-Whitney and Kruskal Wallis rank sum) were used to analyze differences between different conditions. Cronbach's alpha was used to estimate the reliability of H&E and PHH3 mitotic counts. The relationships between H&E and PHH3 mitotic counts were studied using Poisson-type regression models [21], a class of models needed for dealing with possible over-dispersed count data. Overall survival was defined as the time elapsed from the date of lung cancer diagnosis to the date of death or the last visit. Survival analyses were performed using Kaplan-Meier estimates of survival distributions and survival curves were compared using the log-rank test. Cut-off points for H&E and for PHH3, which divided cases into prognostically different groups, were selected on the basis of the highest statistical significance (lowest P value) on the log-rank test among the groups. Finally, hazard ratios and 95% confidence intervals were estimated by the Cox proportional hazards model for multivariate survival analysis.
